# Anthropo-Mechanical Cradles: A Multidisciplinary Review

**DOI:** 10.3390/ijerph192315759

**Published:** 2022-11-26

**Authors:** Maciej Sydor, Jessica Pop, Anna Jasińska, Marek Zabłocki

**Affiliations:** 1 Faculty of Forestry and Wood Technology, Poznań University of Life Sciences, 60-637 Poznań, Poland; 2 College of Health Sciences, Midwestern University, Downers Grove, IL 60515, USA; 3 Faculty of Civil and Transport Engineering, Poznan University of Technology, 60-965 Poznań, Poland

**Keywords:** cradle, human health, energy, monitoring of physical parameters, sway, cradleboard, swing, bassinet, baby, infant, furniture, furniture design

## Abstract

Domestic cradles are beds that are movable but non-mobile for babies up to five months of age. The “anthropo-mechanical” cradle simulates the physiological movement of the human body. The article reviews scientific literature discussing the impacts of swinging on infants, provides classifications of all currently used cradles due to how the child moves, and briefly describes modern technologies within cradle automation. This made it possible to calculate and propose safe motion parameters within mechatronic cradles. The main conclusions of the article are as follows: (1) the scientific literature reports the beneficial effects of harmonic movement on a child, (2) motion analyses substantiating the classifications of all cradles into six types (tilting, yawing, hammock, Sarong, swing, and surging cradle; the classification criterion included the nature of the cradle movement in relation to the planes and anatomical axes of the child’s body), (3) modern technologies allowing for the use of movement with thoughtful parameters, thus, safer for a child, (4) movement within the parameters similar to the motion and speed passively performed by the child in the womb while a mother is walking was considered beneficial and safe, and (5) the use of advanced technology allows for the possibility to devise and create an automatic mechatronic cradle with a child-safe motion. Future innovative anthropo-mechanical cradles that follow physiological human motion parameters can be used safely, with a vertical amplitude ranging from −13 to + 15 mm and a frequency of up to 2 Hz.

## 1. Introduction

A well-designed domestic cradle for babies should be technically feasible, meet aesthetic requirements, offer adequate functionality, and be safe to use. The technical feasibility of a cradle, like any other piece of furniture, is achieved by a properly prepared technical design [1,2,3,4,5,6]. Furniture aesthetic design quality, including cradles, depends on many subjective factors, such as culture, fashion, and personal sense of aesthetics [7,8]. A significant problem in the design of the cradle is effectively “incorporating” all human factors into the product. The application of psychological and physiological determinants to the products include human error resistance, proper functionality, enhanced safety of use, and comfort [6].

Cradles can pose a threat to children and their safety. Falling out of the cradle has the highest injury risk rate for children ages 6 to 8 months [9]. The materials used for cradle construction, wood, wood-derived materials, metals, plastic, adhesives, varnishes, and their emissions could pose a danger when used, such as improper cradle operation due to missing elements, overuse, or improper assembly. Although different furniture materials could be used in cradles, the significance of using safe materials is high since infants or babies are involved. The design requirements for cradles are listed in the EN 1130: 2019, ASTM F 2194–16e1: 2016, and ISO 2631-1: 1997 standards. These standards hold the minimum requirements for rigid element spacing, static loads, stability, foam pad thickness and dimensions, side height, mattress flatness, rocking angles, prohibit the use of accessible small parts, sharp edges, or self-assembly components, and include appropriate usage information.

The primary function of a cradle is to soothe a baby with harmonic movements. Classic cradles are propelled by human muscle strength with a rocking motion where the amplitudes and frequencies are intuitively selected by the child’s caregiver, whereas mechatronic cradles perform movements automatically without regard to the parameters of human motion. The term “anthropo-mechanical” can be defined as a combination of the terms “anthropometric” and “biomechanics”, where we utilize the principles of physiological characteristics for measurement and the mechanical properties of motion produced within the human body. It is not a very common word, but it nicely conveys the desired meaning and is used in the scientific literature [10].

This article assumes that it would be best to reproduce the baby’s movement in the womb and use these parameters within cradle movement. Therefore, it is justified to conduct an interdisciplinary review of the literature and techniques on the impact of rocking a child and the types of modern cradles (with a particular emphasis on automatic cradles). A compact review of the literature on the kinematics of a pregnant woman’s gait, conducting primary kinematic analyses based on literary information, and proposing innovative construction of cradles may be implemented within anthropo-mechanical motions following designated parameters.

## 2. The Influence of Rocking on the Baby, a Brief Review of the Literature

Rhythmic movement improves sleep quality [11]. This is due to stimulation of the vestibular apparatus [12]. The vestibular apparatus begins to form on day 30 of embryonic life, and by day 49, its morphogenesis is complete [13]. At this time, the fetal vestibular system is stimulated when the mother moves, and this positive effect also takes place after birth. Clark et al. noticed accelerated reflex maturation after vestibular stimulation of term infants [14]. Vestibular stimulation by horizontal rocking is used therapeutically in human preterm infants [15]. Clinical trials showed that rocking cradles or oscillating waterbeds reduced the frequency of pauses in breathing, known as apneic spells [16]. The benefits of rocking on the youngest children are summarized in Table 1.

Chisholm and Swaddling (1978) presented the results of an ethnological study of the use of cradles by Navajo Indians. According to the results, cradle use decreases from about 16 h a day in the first three months of a child’s life to less than 9 h before his first birthday. Compared to European babies, Navajo babies spend significantly more time in actual or potential social contact with adults. The author suggests that the cradle can be used in Western cultures to reduce the suffering of infants deprived of social contact [17]. Rhythmic rocking of babies is essential for developing the neuromuscular system, as it affects motor development. Thelen (1980) suggests that vestibular stimulation deficiency may be one of the causes of persistent motor stereotypes in infants (persistent stereotypes, code F.98.4 in ICD-10) [18].

Bayer et al. (2011), investigated the relationship between rocking and sleeping [20]. The conclusions of their study indicated that lying on a slowly swaying bed (0.25 Hz) eases falling asleep and preferably extends the duration of the N2 sleep stage (one of three non-rapid eye movement (NREM) sleep phases before entering the REM period). During this stage, the child will be at the beginning of a deep sleep. The results of the research from these authors can lead to confirmation of the belief that rocking a child supports healthy sleep.

Rocking can also be therapeutic for premature babies. Tuck et al., in 1982, stated that rocking prevents bouts of sleep apnea in premature babies and developed an incubator that is moved by pneumatic actuators and sways in different planes [16]. In 1982, Pierpont and Kramer conducted studies based on the simulation of selected features of the intrauterine environment. Premature babies were placed on waterbeds, gently rocked, and exposed to auditory stimuli. As a result of the research, it was confirmed that the premature babies rocked on a waterbed were more likely to eat and had increased body weight faster, compared to the non-rocking control group [23].

Some authors present research results on the harmful effects of rocking on infants in the cradle. These threats are summarized in Table 2.

The use of the cradles can also be life-threatening due to sudden infant death syndrome (SIDS) occurring in infants and young children left to sleep unattended. Byard, Beal, and Bourne (1994) analyzed 30 cases of accidental asphyxia and described two deaths resulting from constant tilting of the cradles [24]. Beal et al. (1995) experimentally confirmed, by analyzing video documentation, that it is unacceptable to leave a child unattended in a cradle with the possibility of a constant tilt by more than 10°. Infants in a tilted cradle, especially with their hands trapped between the torso and rungs, or with their hands extended beyond the rungs, could not obtain a fully open airway, resulting in death [25]. These observations are also confirmed by Moore et al. (1995), as they analyzed two cases of infant deaths that were several weeks old due to the lack of protection against excessive swinging cradles. In control studies that involved observation of other children, a substantial risk of suffocation due to positional asphyxia was confirmed. Automatic asphyxiation was the most probable cause of death in the two analyzed cases [26]. Another case of a slightly older 11-month-old infant, resulted in death from suffocation due to body affixation, as described by Saha, Batra, and Bansal [28]. Ackerman and Gilbert-Barness (1997) described 15 cases of cradle-infant affixation, 10 of which were fatal. In all cases, the cradle was tilted permanently by an angle greater than 5 degrees [27].

The use of bassinets can also pose a risk of children falling out. Falls from the changing table, the arms of the caregivers, and out of bed cause the most infant injuries [32]. Warrington, Wright, and Team have reported the results of an extensive study of accidents in children up to 6 months of age (2001). Parents of 11,644 babies were surveyed. There were 3357 accidental falls out of 2554 children, 53% fell off the bed or couch. Only 14% reported visible injuries; 97% of visible injuries concerned the head. Only 21 (<1%) falls resulted in concussions or bone fractures [36]. Lyons and Oates (1993) reported 124 falls out of bed (including cradles) and 84 from the bed. They found superficial injuries in 29 cases, and a skull fracture in one case [30]. Sarong Cradles, typical in the Asian region, are acknowledged by the risk of a child falling out. Ng et al. (1997) analyzed 19 injuries caused by a child falling out of the cradle. Data were collected for 9 months based on interviews with the children’s caregivers. The ages of the 19 children ranged from 13 days to 29 months. Accidents occurred while the children were sleeping (14), playing (4), or feeding (1). Although most of the head injuries sustained from falls have been minor, there is a risk that such accidents may lead to more severe injuries [34].

Table 1 summarizes the beneficial effects of rocking on an infant’s development. The rhythmic rocking in the cradle positively affects the development of the infant’s brain. This movement stimulates the vestibular nervous system inside the brain stem and interacts with the cerebellum and the inner ear mechanism. Babies stimulated via systematic swinging have a better appetite and faster development, e.g., they gain a regular sleep cycle earlier, and sleep is calmer compared to children not stimulated by harmonic movement. However, using a cradle ith a motion mechanism can potentially harm the infant (Table 2). Thus, the literature, do not only points exclusively beneficial of using a cradle with a drive but also suggests some dangers detrimental to the infant’s health. The design of an automated, safe-to-use cradle is paramount to receive all the possible benefits and pose absolutely no danger.

## 3. The Proposed Categorization of Cradle Types Based on State of the Art and Types of Cradles

**Figure 1:**
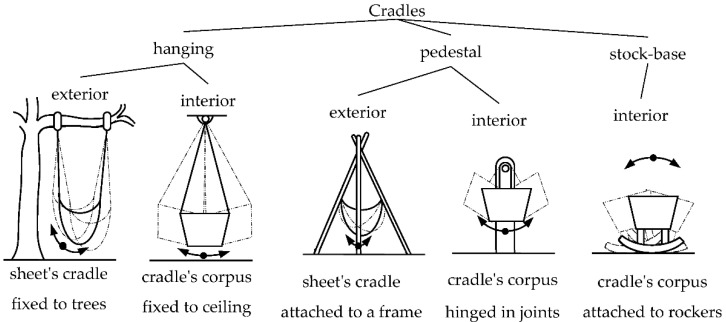
Types of the most common cradles in the 19th century and the first half of the 20th century (inspired by [37]).

Understanding how cradles are typically designed is crucial to understanding how to improve existing designs and potential automation. Veselovský and Baďura analyzed the history of the development of cradle forms and distinguished three main categories: hanging cradles, pedestal cradles, and base cradles. The hanging cradles were hung inside or outside of the rooms. The pedestal cradles consisted of two subassemblies, a fixed pedestal, and a movable bed, while the base cradles had a “single-body” structure [37]. The typology of cradles used in the 19th and the first half of the 20th century, according to Veselovský and Baďura, is presented in Figure 1.

**Figure 2:**
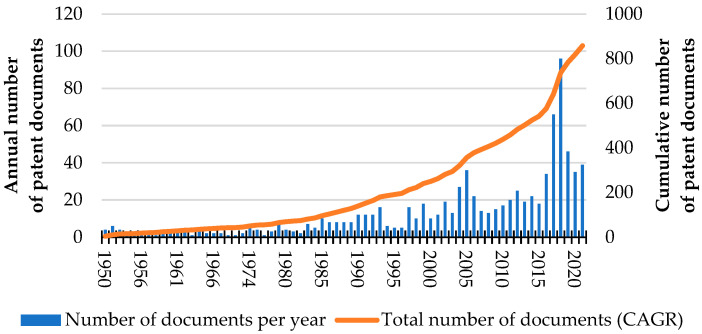
The number of patents documents published between 1950 and 2021 with CPC classification code A47D9/02 (cradles for babies).

Contemporary cradles are furniture of various structures. There have been 967 patent documents filed within patent office databases relating to cradles for babies (TAC “cradle” and CPC classification code A47D9/02, searched by The Lens (Query: “class_cpc.symbol:A47D9\/* AND (title:(cradle) OR abstract:(cradle) OR claim:(cradle))”, range 1950–2021)). They represent 722 patent families. Figure 2 shows the annual increase in the number of patent documents related to cradles for babies.

**Figure 3:**
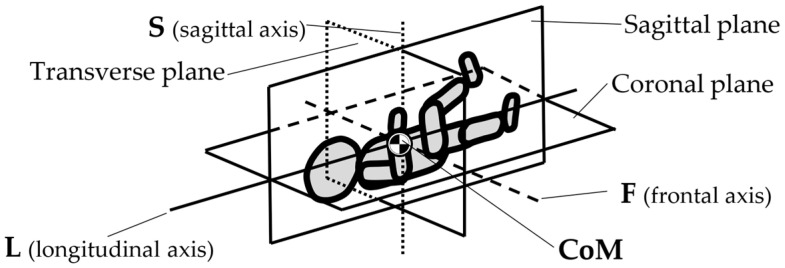
Anatomical planes and axes with the child’s Center of Mass (CoM) location.

However, inventions do not always become genuine products. Therefore, the review and analysis of the furniture market would provide a strong basis for the constructional classification of cradles for children. The most useful from the point of view of mechanics is the classification of cradles related to the anatomical axes and planes of the human body. Anatomical planes introduced and named by Anderson (1892) [38], and anatomical axes are shown in Figure 3.

The cardinal planes divide the body into equal portions. The cardinal sagittal plane (frontal axis) divides the body into the right and left parts. The cardinal coronal/frontal plane divides the body into anterior and posterior parts (sagittal axis). The cardinal transverse (horizontal) plane divides the body into superior and inferior parts (longitudinal axis). The axes of the body are related to the cardinal planes.

A review of state of the art in the field of cradles combined with the anatomical planes and reference axes allows for proposing a new classification of cradles. The classification of cradles, with photographs and characteristics of physical movement, is presented in Table 3.

In Table 3, a typical notation of the anatomical planes and directions of the human body is used, as shown in Figure 3. Table 3 shows that there are six types of cradles: (a) tilting—tilts forward and backward because it has two transverse rockers, (b) yawing—swivels left and right because it has two longitudinal rockers, (c) hammock cradle, (d) sarong cradle (or one pendulum cradle), (e) swing cradle (or two pendulum cradle) and (f) surging or four-pendulum cradle. According to the classification proposed in Table 3, cradles supply different possibilities for setting the human body in motion. They allow movement in selected planes and rotate along chosen body axes. This is summarized in Table 4.

As shown in Table 4, the most common cradles (a–c) offer two independent movements (having two degrees of freedom). The Sarong cradle (d) is deemed the most dangerous [34], with up to five degrees of freedom. The e-type cradle, suspended on two handles, has three degrees of freedom, plus one degree highly restricted. On the other hand, the least mobile is the four-pendulum cradle (f), which can move only in one plane and one direction.

The presented analysis shows that more degrees of freedom of the cradle’s corpus cause the highest risk of accidents (based on the Sarong cradle) and lead to an increased risk of a childs overstimulation.

## 4. Mechatronic Cradle Designs in the Scientific Literature

Many hours of rocking the cradle are arduous, and attempts have been made to mechanize this activity; an early example may be the invention of Marie R. Harper, La Mirada, and Maxine R. Blea, “Automatically rocking baby cradle”, US 3769641, 1973, or the invention of Gim Wong, “Automatic baby crib rocker” US 3952343, 1976. These hnological developments made it possible to construct mechatronic cradles, which relieve the parents of the child and eliminate the risk of spontaneous suffocation of a child left unattended in an excessively tilted cradle [26,28]. The system for automatic monitoring and correction of the child’s body position, as described by Sudharsanan and Karthikeyan (2013), may be helpful for this purpose. The authors proposed placing an accelerometer on the child’s forehead to monitor its body position while sleeping. If an incorrect body position is detected, two servomotors can automatically correct it by tilting the cradle in the proper direction. Therefore, this cradle is designed to prevent SIDS [29] automatically.

Offering solutions based on the Internet of Things (IoT) is relatively popular within the literature. With the developments of computer science, automatic adaptive systems are proposed using various sensors: sound, a child’s body temperature, the environment, air humidity, heart rate, or the amount of carbon dioxide in the exhaled air. Some sensors can also detect unusual baby movements or incorrect positions. The alarm signal can be generated by software that detects irregularities; the mechatronic cradle executive systems will activate and adjust the rocking parameters of the cradle, while the data acquisition system, using cloud techniques, archives the sensor readings. One of the first systems to detect and identify the types of crying from a baby was proposed by Chau-Kai-Hsieh and Chiung Lin, with the title of their invention filed in 1997 as “Baby Cry Recognizer” US 5668780. Table 5 lists numerous scientific publications describing the various concepts of remote supervision of automatic cradles.

Table 5 shows that the number of scientific publications describing mechatronic cradle systems has increased in recent years. In addition to these publications, there have been attempts to reproduce the rocking movement in the mother’s arms through a mechanized cradle. They focused on the motion analysis of a mother’s embrace while rocking a baby, the development of an excitation apparatus [45], and the rocking motion of the baby sleeping on the mother’s lap [37]. A common feature of the systems described in Table 5 included the detection and appropriate classification of the types of crying from a baby. Based on this, the device can run at an appropriate operating program [59,60,61,62].

**Figure 4:**
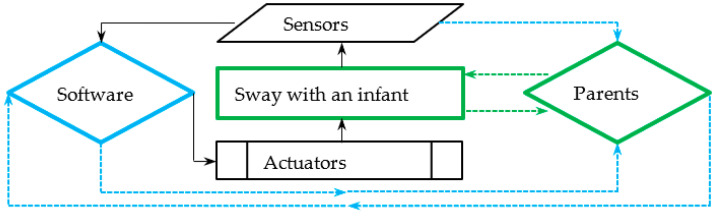
The information flow diagram in mechatronic cradles (summary of Table 5).

Figure 4 shows the operation scheme and data flow structures connected to the mechatronic cradles described in the scientific publications. Each cradle was equipped with actuators that put the cradle in sway mode. Built-in sensors monitored the temperature, air humidity, sound, body position, and mattress moisture. With the help of cameras, a video was transmitted directly to the parents’ devices such as their smartphones or laptops. The collected data was automatically sent to the system, which, thanks to the constantly updated data, was able to change the sway mode of the child or send a message to the parent at the time of danger. At the same time, after receiving information from the system, the parent could decide to introduce or not introduce changes in sway mode, turning on a light, fan, or calming music. A flow diagram of the mechatronic cradles described in Table 5 is shown in Figure 4.

The center of the diagram shown in Figure 4 represents the sway of an infant. The sway is set in motion through actuators. The parameters of the cradle’s movement and the baby’s current needs are read by sensors, and the entire system’s operation is managed by software. Parents can directly or indirectly supervise the work of the described system and the baby directly or remotely using its sensors (video, sound, and others). They can adjust the software signals by interrupting or changing their operations. The black arrows indicate the automatic flow of information between the mechatronic cradle and (IoT) elements. Blue arrows indicate the flow of information between the mechatronic devices, and the parents. Green arrows indicate the flow of information between the child and the parents.

## 5. Kinematics of the Gait of a Pregnant Woman: A Brief Review of the Literature and Basic Calculations

A fetus in intrauterine life is stimulated kinematically by the gait of the pregnant woman. The gait of a pregnant woman is modeled by changes in the position of the center of mass (CoM) within her body while walking at a comfortable speed, the so-called physiological. The first step in evaluating gait is to establish the value of the velocity of various body heights in the advanced pregnancy stage. Knowing the velocity value produced during walking makes it possible to compute the parameters of harmonic movement (amplitude and frequency) concerning the body’s center of gravity. Attaining these parameters allow ilding a cradle with a mechanically forced motion corresponding to the “anthropo-mechanical” parameters. According to Gedliczka and Pochopień, nonpregnant women between the ages of twenty and forty, with a height of 1.61 m, had an average speed of 1.49 m/s while walking [63]. The angular range of motion within the joints of the lower limbs of a pregnant woman did not change significantly when compared to the studied control group; however, a change in the length of stride was observed by 3 to 4 cm shorter than the control. [64]. The average total length of a pregnant woman’s right and left steps (in the third trimester) was 1.247 m, and the duration of one gait cycle was 1.086 s [65].

**Figure 5:**
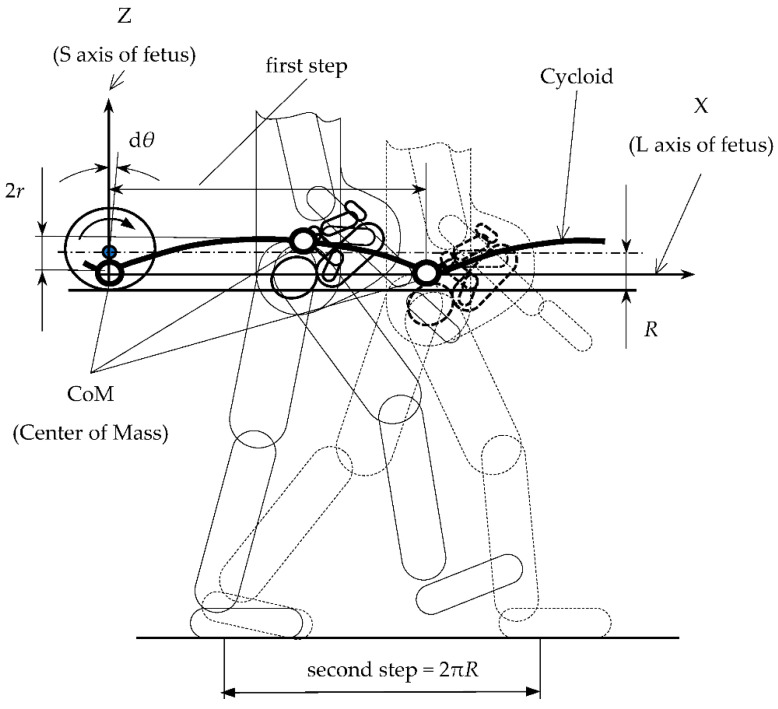
The center of mass (CoM) of a walking pregnant woman with a fetus.

The inverted pendulum model, a standard human gait model, can determine the approximate characteristics of the movement of the center of mass within the examined body [66]. This movement follows a roulette described by the trochoid equation or a specific trochoid like a cycloid. The epicycloid represents the path of a point at the end of the radius on a wheel that rolls without skidding on a flat surface. Figure 5 depicts a graphic representation of this movement.

The cycloid is defined by two parametric Cartesian equations: x=R · θ−r · sin(θ) and $z=z_{0}-r\;\cdot \;cos\left(\theta \right)$. In the equations, *θ* is the angle between the radius of the circle and the vertical direction, *R* is the radius of the circle, *r* is the distance of the point attached to the radius from the center of the circle, and *z_o_* is the height of the center of the circle. The variables x and z define a point’s coordinates attached to the radius. The first derivative of the trajectory *θ* corresponds to the angular velocity *ω* of the cycloids [66].

**Figure 6:**
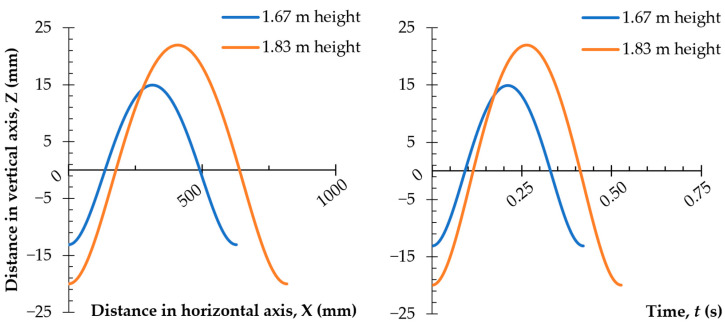
The calculated changes of the center of mass (CoM) for two women with different heights: dependent on the distance traveled and time.

Considering the data provided by Carpentier [66], a graph on the dependence of the vertical coordinate on the horizontal coordinate, at the center of gravity of two women, with two body heights was prepared (1.67 m and 1.83 m—Figure 6). The dependence of the change in position of the center of mass (CoM) in time is shown in Figure 6. The graphical interpretation of the cycloid equations based on the adopted constants is showed in Table 6.

The graphs presented in Figure 6 show that in a woman with a height of 1.67 m, whose step is about 600 mm, the coordinate position of the center of mass varied from −13 mm through +15 mm. This change occurred in 0.48 s, which translates into movement of the center of mass (CoM) at a frequency of about 2 Hz. The presented relationships make it possible to design the trajectory of cradle movement to correspond to the spatial positioning of the pelvis. The cradle could be equipped with a mechanism allowing for frequency fluctuations to be programmed according to the natural swing cycle of the baby in the womb while depending on the woman’s height and changes in the position of her body’s center of gravity.

**Figure 7:**
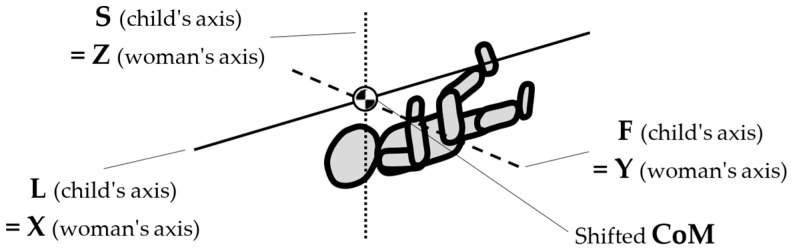
Shifted arrangement of child axes and anatomical planes.

To map the changes in a walking woman’s CoM, and the child’s movement, the planes and anatomical axes of the child’s body within the cradle should be shifted so that their zero point coincides with the CoM of the pregnant woman. The shifted system of the anatomical childs axes is shown in Figure 7.

**Figure 8:**
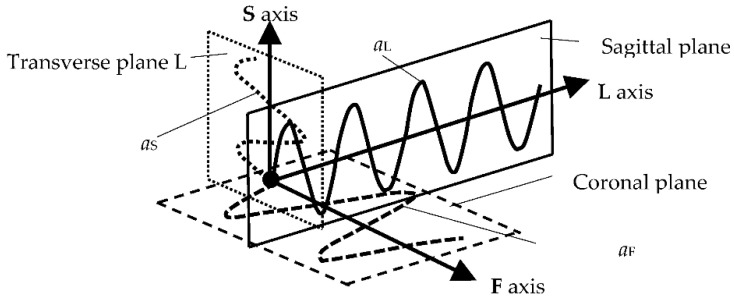
Schematic representation of the action of the accelerations *a*_L_, *a*_F_ and *a*_S_ acting in the three axes L, F, and S on the child during the mother’s gait.

The characteristics of the anthropo-mechanical cradle motion are shown in Figure 8. Schematically shown elements of the acceleration, within the mother’s gait, regarding her center of mass (CoM), *a_L_*, *a*_F_, and *a*_S_, are acting in three orthogonal axes, L, F, and S (X, Y, and Z). This figure was prepared based on data provided by Jansen et al. in Figure 1 in the reference [67] and in association with Figure 3, Figure 5 and Figure 7 in this paper.

The following insights for the design of anthropo-mechanical cradles are as mentioned:

1. When selecting the parameters of the movement of the anthropo-mechanical cradle, apart from the amplitude in the vertical axis (*Z* axis in mm) and the frequency (in Hz), it is worth taking into account the remaining parameters of the dynamics of the movement of the woman’s center of mass (CoM) while walking, i.e., amplitudes and accelerations acting on the three anatomical axes of the body (L, F, and S).
2. At each tilt, the directions of the accelerations *a*_L_, *a*_F_, and *a*_S_ change in the cradle. The direction of the CoM acceleration vector changes only for the S (vertical) and F (transverse) axes during the mother’s gait. For the L axis, the direction of the acceleration vector is constant. Thus, the resultant movement of CoM is quite complex. It takes place simultaneously within the three axes L, F, and S. The challenge would be to obtain a cradle movement where the position of the fetus during pregnancy and the parameters of the physiological gait of a given mother would be correlated. It would then be possible to program the cradle to adjust to each child individually and imitate the movement of being in its mother’s womb. It seems that typical cradles (Table 3) do not best reflect the movement of the CoM within a pregnant woman’s gait. Therefore, a new solution for the cradle structure should be proposed.
3. The frequency of the cradle’s motion must not exceed the resonant frequency of the baby’s organs, as it could pose a danger to the child’s health. Fortunately, these frequencies are larger than the postulated range (up to 2 Hz). As is known, the resonant frequency of the human body ranges from 5 to 10 Hz—depending on the individual’s body structure and position—lying, standing, or sitting [68]. However, excitations of a lower frequency can stimulate the abdominal organs to experience strong vibrations [69].

The recommended rocking parameters of a child include an amplitude (*A*) of 28 mm and a frequency (*f*) of up to 2 Hz, which are confirmed in the literature. Byrne et al. studied the effects of rocking on 36 full-term infants (24–72 h of age) on the experimenter’s shoulder. When they used horizontal rocking movements, with 30 cycles per minute (*f* = 2 Hz) and a duration of up to 3 min, they obtained the best effects of calming the child [70]. Peak acceleration level (*a*_max_) may be computed by using the formula: $a_{\max}=\frac{4\pi^{2}\;\cdot \;f^{2}\;\cdot \;A}{32}$ (m/s^2^). Therefore, the recommended rocking motion parameters mentioned above result in *a*_max_ = 0.07 m/s^2^. Vrugt and Pederson recommend *a*_max_ values between 0.025 and 0.075 m/s^2^ [71].

Inappropriate motor stimulation, such as inharmonious shaking or rocking, can be fatal or cause permanent disability. Shaking can cause complications such as brain damage, cerebral palsy, hearing loss, blindness, learning difficulties, seizures, and paralysis [72]. Babies between the ages of 2 and 4 months are most at risk of shaking injuries. Accidental stimulation leads to an emotional disturbance in the child. Rhythmic stimuli allow for the synchronization of rhythms, leading to the facilitation of selective attention and the learning of perception [73]. At the time of birth, significant environmental changes occur, differing from what life has been in the womb of the baby’s mother. Increasing attention has been paid to environmental and ectopic changes, indicating a significant role in developmental and behavioral outcomes such as optimal growth and development [74].

The calming effect of walking by the mother on children was reported in the literature. Esposito et al. monitored the responses of 12 healthy infants aged 1–6 months. The scientists wanted to compare holding a crying baby with carrying an infant while walking for 30 s or longer. The study found that the babies that were carried were more relaxed and soothed than the babies whose mothers sat and held them. Thus, maternal walking is more effective in calming infants than other non-rhythmic motions [75]. Infant carrying is a biological norm for human caregiving and results in a carrying-induced calming response for the child [76]. Carrying during walking is a well-known intuitive and multicultural mother’s reaction to a baby crying. The effectiveness of this effect has been confirmed by scientific research [77]. Therefore, using motion parameters in the cradle that resemble the walking mother positively affects the infant without the risk of overstimulation.

## 6. Conclusions

The harmonic movement has a beneficial calming effect on normal developing babies and a beneficial therapeutic effect on premature babies. The literature cited in Section 2 of the article highlights that constant stimulation of babies’ vestibular system due to systematic rocking improves development and the formation of a restful and consistent sleep cycle. Thus, rocking allows for the transition from being awake to sleeping easier. However, using cradles could increase the risk of suffocation and injury if a limb gets stuck or the baby falls out. Therefore, cradles should meet the minimum requirements described in the standards cited in the Introduction.

Mechatronic cradles that mechanize a rocking movement have been described in the literature (usually having an added remote supervision function). However, such devices can be dangerous and may have excessive movement on the child. The information and calculations within the cited literature made it possible to use physiological movements that do not increase the risk of overstimulation; therefore, the parameters of this movement were calculated, and five innovative concepts of cradle design were proposed. The review of the literature and own analysis described in this study suggest that:

1. The harmonic movement has a beneficial effect on babies; in particular, rhythmic rocking of the cradle has a positive impact on the development of an infant’s brain and a beneficial therapeutic effect on premature babies. Cradle rocking can naturally link a baby’s two stages of life: when the fetus is rocked in the womb by the mother’s motion of locomotion and the period of independent walking.
2. A crucial component of a cradle’s design involves the safety requirements to prevent falls and entrapment. To avoid the risk of the child falling out: footholds in the cradles are not allowed, minimum side heights are strictly defined, and the maximum age of a child sleeping in the cradle is five months. An excessive tilt of the cradle greater than 10° should be avoided to prevent strangulation or suffocation risks.
3. The multidisciplinary literature review and kinematic analyses suggest that cradles should be classified from the point of view of the possibilities and limitations of a child’s rocking motion parameters and not from a view of the external forms. That is why we proposed a classification of cradles into six types, offering from 2 to 5 degrees of freedom in various configurations (tilting, yawing, hammock, Sarong, swing, and surging cradle).
4. Modern technology enables the design of cradle movement to include more thoughtful parameters and ensure the safety of the child. This includes calculating the approximate movement characteristics of the center of mass within the human body while in motion, as well as designing the limits of a cradle’s tilt to correspond with the directions and parameters of the spatial positions of the pelvis during human gait patterns.
5. Compared to mechanically driven cradles, it is preferable to use “anthropo-mechanical” cradle movements to prevent a child from being overstimulated. Child movement in an “anthropo-mechanical” cradle should be maintained within the parameters of the passive motion, and speed performed within the mother’s womb while walking can be considered safe. The movement parameters in such a safe cradle should be determined based on the mother’s height. According to our analysis, safe cradle movements could be achieved by having a vertical amplitude ranging from −13 to +15 mm and a frequency of up to 2 Hz. The frequencies of the cradle’s movement must not coincide with the resonant frequencies of the baby’s organs, as it can pose dangerous effects on the child’s health.

## Figures and Tables

**Table 1:** Positive Impact of Rocking.

The Range of Tests	The Confirmed Influence of Rocking on the Child	Reference, Year, and Number of Citations in Scopus
The development of healthy children	Rocking has a positive effect and replaces social contacts	[17], 36
	A rhythmical stereotypy is beneficial for infants	[18], 60
	Vestibular, kinesthetic stimulation has a positive homeostatic effect	[19], 5
	Rocking is beneficial during a short nap	[20], 66
	It is unclear whether rocking has any long-term benefit or harm for the infant. However, it is used in many cultures	[12], 0
Therapeutic effect on premature babies	Gently oscillating water beds reduce apnea in premature babies	[21], 62
	Rocking prevents sleep apnea attacks in premature babies	[16], 23
	The kinesthetic stimulation is used at treating sleep apnea of prematurity	[22], 19
	Rocked premature babies were more eager to eat and increased body weight faster	[23], 77
	Vestibular stimulation (rotatory and torsion swing test) supports the sensorial maturation of Small for Gestational Age infants	[13], 7

**Table 2:** Threats of rocking.

The Type of Threat	Consequences of the Threat	Reference, Year, and Number of Citations in Scopus
Danger to life	Suffocation caused by a cradle tilted greater than 10 degrees or too soft a mattress	[24], 1994, 75 [25], 1995, 10 [26], 1995, 18 [27], 1997, 10 [28], 2010, 10
Health hazard	Falling out of bassinets (including cradles)	[29], 1987, 137 [30], 1993, 151 [31], 2000, 34 [32], 2016, 13 [33], 2021, 0
	Falling out of Sarong’s cradle	[34], 1997, 4
Overstimulation	A mechanical rocking bed could increase withdrawal symptoms in drug-affected infants	[35], 1999, 27

**Table 3:** Types of cradles and directions of the infant’s body movement resulting from their construction.

(a) Tilting Cradle	(b) Yawing Cradle	(c) Hammock Cradle—Two Variants:	
		(c1)	(c2)
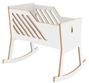 (Tuli cradle, Nuki, Poland)	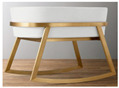 (Caden bassinet, RH, USA)	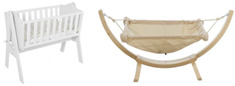 (LEA, Mamaania, Poland) (Kaya Natura, Frommummy, PL)	
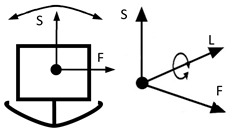	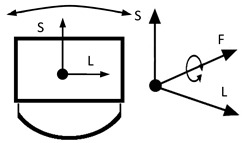	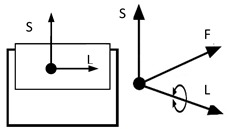	
**(d) sarong cradle or one pendulum**	**(e) swing cradle or two pendulums**	**(f) surging cradle or four pendulums cradle**	
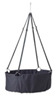 (Leander Hanging Baby Cradle, Cuckooland, Great Britain)	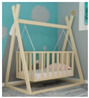 (Montessori Bed, Montessori Bed Plans, n.d.)	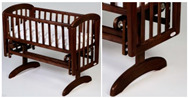 (Anna Glider, Troll Nursery, Latvia.)	
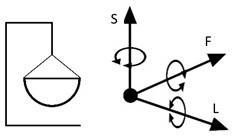	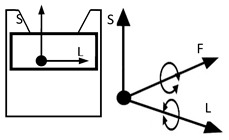	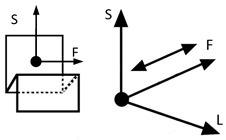	
Notation: F—frontal axis, L— longitudinal axis, S—sagittal axis (according to Figure 3)			

**Table 4:** The type and number of degrees of freedom for each type of cradle.

	Longitudinal Axis (LA)	Sagittal Axis (SA)	Frontal Axis (FA)	Transverse Plane (TP)	Sagittal Plane (SP)	Coronal Plane (CP)	No. Degrees of Freedom (DoF)
Cradle a	✓	(–)	(–)	✓	(–)	(–)	2
Cradle b	(–)	(–)	✓	(–)	✓	(–)	2
Cradle c	✓	(–)	(–)	✓	(–)	(–)	2
Cradle d	✓	✓	✓	✓	✓	(–)	5
Cradle e	✓	(–)	✓ ^(–)^	✓	✓ ^(–)^	(–)	3 + 1 ^(–)^
Cradle f	✓ ^(–)^	(–)	(–)	(–)	(–)	✓	1 + 1 ^(–)^

Notation: ✓ – movement in this anatomical plane or axis is possible, (–) – movement in this anatomical plane or axis is impossible, ✓^(–)^ movement in this anatomical plane or axis is possible, but significantly limited.

**Table 5:** Swing Automation Systems in Mechatronic Cradles.

Bibliographic Reference, Publication Year and No. of Citations in Scopus	The Essence of the Concept	
[39], 1997, 2	Pendulum swinging system. Swing parameters are in the range of 0.4–1 Hz and 0.36–3.6°	
[40], 1998, 0	A swinging system with a noncontact magnetic driving force and various programmable swinging patterns. The band of frequency is limited from 0.4 Hz to 1 Hz, desired swing amplitude of ±0.0018 rad (±0.1°)	
[41], 2000, 2	A “static” (nonadaptive) system of a remote swing program selection. Swing parameters within the range 45–50 cycles/min. were used (0.75–83 Hz). The system should not be operated for more than 30 min.	
[42], 2009, 1	Motion sensors, a microphone for recording a baby’s cry, and a system that performs the movement with parameters selected by the algorithm. The cradle movement limits have not been specified.	
[43], 2011, –	A system detects the baby’s crying, then activates swinging and colored lights. Cradle movement limits the use of parameters that have not been specified (San Jose State University project, not indexed in Scopus).	
[44], 2013, 4	A real-time baby sleeping position monitoring and correction system to avoid SIDS (the cradle motors prevent the baby against to sleep in its prone position). Cradle movement limits parameters have not been specified	
[45], 2015, 6	Electrically driven cradle with infant cry recognition. The cradle drive uses resonance to reduce energy consumption during use. The desired movement parameters were not specified	Automatic “intelligent” rocking programs, e.g., automatic start swinging when the baby cries, if the baby stops crying before the specified time, the cradle will stop if no alarm or message will be sent to baby caretakers.
[46], 2016, –	Electric-powered cradle with baby cry recognition and mattress wet alarm.	
[47], 2017, 1	The mother’s motion while sleeping with her baby on her lap was modeled and re-created in the cradle. A maximum speed of 0.04 m/s was adopted, and 2 m/s^2^ was the starting acceleration of the cradle.	
[48], 2018, 0	The crying detection and classification (hungry, pain, sleepy, non-crying) system saves the collected data on the server and shares them via the mobile application. Three types of cradle operation: rocking, activating calming music, and alarm. The swing motion parameters have not been specified.	
[49], 2019, 0	Infant cry detector and wet mattress sensor, then swinging or sending a message to caregivers. The swing motion parameters have not been specified.	
[50], 2019, 30	Remote supervision of a child in the cradle using a laptop or smartphone. Infant crying detection and automatic swing activation. Supervise the humidity and temperature of the air and turn on the fan if it exceeds 28 °C. Possibility of remote switching on calming music. The cradle movement parameters used are not reported.	
[51], 2020, 0	Automatic rocking when the baby cries (if the baby stops crying before the specified time has elapsed, the cradle will stop). Alarm or information to caregivers if the baby cries for more than a specific time and when the mattress is wet. Remote monitoring of infant body temperature, heart rate, air temperature, and humidity). Incubator for hospital and home use.	
[52], 2020, 0	The web camera, humidity and temperature sensor, cry detector, remote monitoring, and automatic swing system. The swing motion parameters have not been specified.	
[53], 2020, 1	When a cry is detected, the cradle can sway automatically or display a warning light when an abnormality is detected. The swing motion parameters haven’t been specified.	
[54], 2020, 1	The user can control the cradle’s swing in manual mode and start music playing using a smartphone. The cradle detects crying, in the following, swings automatically, plays calming music, and send messages to the designated phone.	
[55], 2021, 0	Android-based, remote monitoring system with a motor to swing the cradle, a cry detector, and a wet mattress sensor.	
[56], 2021, 0	A remote monitoring system with sensors identifying a baby’s cry, body temperature, heart rate, and motion and posture status of the infant. The cradle offers electronic swinging, pleasant sounds, and other features, e.g., switching the fan on or off.	
[57], 2022, 0	The remote monitoring system of a baby cry, body temperature, and wet mattress sensors. A cradle is equipped with pre-fitted air purification system. The cradle works based on the Internet of Things (IoT) principles and offers electronic swinging, pleasant sounds, and other features, e.g., self-activated automated eye-catching moving toys.	
[58], 2022, 0	A machine learning remote monitoring system with baby’s vital signs and ambient parameters sensors. The system automatically swings the infant’s cradle.	

**Table 6:** Data adopted in the cycloid equations.

Height of the Woman (m)	Radius of the Circle *R* (mm)	Distance of the Point Attached to the Radius from the Center of the Circle *r* (mm)	Height of the Center of the Circle *z_o_* (mm)
1.67	100	14	0.91
1.83	130	21	1

The denotations used in the table are in accordance with Figure 5.

## Data Availability

All data, models, and code generated or used during the study appear in the submitted article.
